# Metastatic Melanoma to the Urinary Bladder: A Rare Cause of Visible Haematuria

**DOI:** 10.1155/2024/5516547

**Published:** 2024-03-01

**Authors:** Olawale Ogunremi, Dinelle Sirjuesingh, Aniket Deshpande

**Affiliations:** Department of Urology, Colchester Hospital, East Suffolk and North Essex NHS Foundation Trust, Colchester, UK

## Abstract

Bladder metastasis from cutaneous melanoma is a rare pathology. A 79-year-old woman presented to the haematuria clinic on account of painless visible haematuria. Ten years prior to this index presentation, she was diagnosed with melanoma on her right thigh following a total excision of the skin lesion. Cystoscopy showed a pigmented bladder tumour, and the histology report following a transurethral resection was consistent with metastatic melanoma, and further imaging revealed metastasis to the lungs, adrenals, and lymph nodes.

## 1. Introduction

Melanoma is the fifth most common cancer in the United Kingdom and has been established as the deadliest of the skin cancers [1]. It may metastasize to any part of the body; however, metastasis of melanoma to the urinary bladder is a very rare clinical finding. Melanoma metastases to the urinary bladder are often associated with synchronous metastases and portend poor prognosis [2–5]. There is possibly an underestimation of this disease due to the patients being asymptomatic as the postmortem series of melanoma patients have found an 18-37% rate of metastases to the urinary bladder [6, 7]. Here, we present a case of metastatic melanoma to the bladder treated with transurethral resection of the bladder tumour and immunotherapy.

## 2. Case Presentation

A 79-year-old female was referred to our haematuria clinic with complaints of intermittent painless visible haematuria of 2-month duration. She noted a progressive right groin swelling which was painless and nonreducible of 4-month duration. She had an excision of a right thigh melanoma with a negative margin 10 years ago. She had no comorbidity, never smoked tobacco product, or drank alcohol and has no family history of cancer.

**Figure 1:**
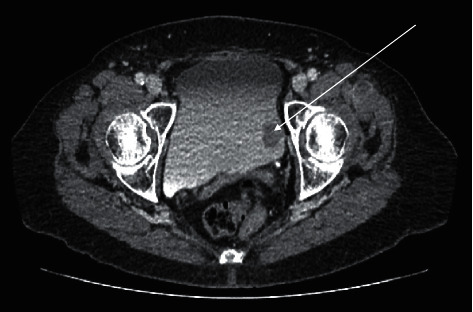
Axial section of CT urogram showing a filling defect on the left bladder wall (white arrow).

**Figure 2:**
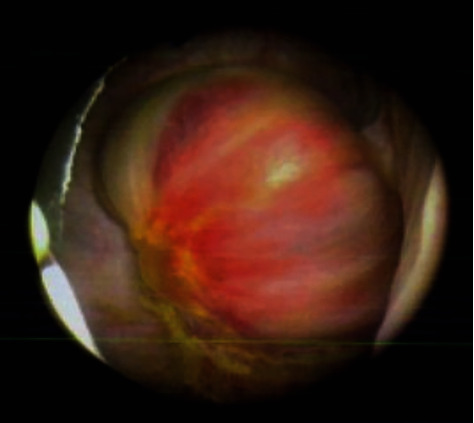
Cystoscopic image showing the left bladder wall tumour.

She had a flexible cystoscopy which revealed a pigmented solid lesion in the left posteroinferior bladder wall. A computed tomography urogram revealed an intravesical 2.4 by 1.5 cm soft tissue mass on the left lateral wall of the urinary bladder (Figure 1), multiple basal lung nodules, bilateral hypodense adrenal masses, retrocrural and 3.4 cm right inguinal lymph node, and a suspected peritoneal nodule. Prior to the transurethral resection, a bimanual examination showed no pelvic mass or thickening. Rigid cystoscopy showed an ovoid, smooth-surfaced pedunculated tumour following which a high index of suspicion of metastatic melanoma was made (Figure 2). A transurethral resection of the tumour was performed, and the core of the lesion was haemorrhagic. A chest-computed tomography scan revealed multiple pulmonary metastatic nodules as well as mediastinal lymphadenopathy, but a magnetic resonance imaging of her brain showed no evidence of any brain metastasis.

**Figure 3:**
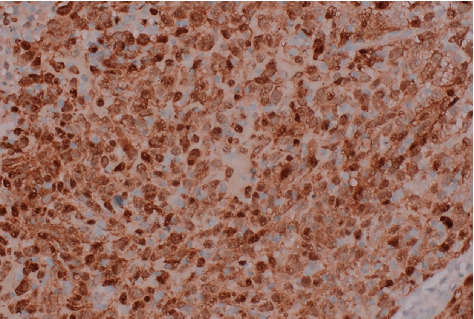
Stain positive for S100 (×400).

**Figure 4:**
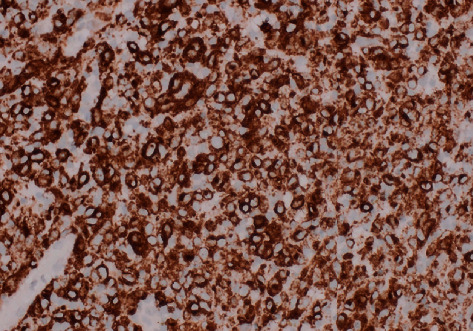
Stain positive for Melan-A (×400).

Histology showed a tumour formed of poorly cohesive sheets and pseudopapillary structures composed of large atypical cells. Immunohistochemically, the cells show staining for S100 protein (Figure 3) and Melan-A (Figure 4) with negative staining for cytokeratin AE1/AE3, p40, and GATA-3, and the appearances are consistent with metastatic melanoma. At the urooncology multidisciplinary meeting, she was referred to the clinical oncologist, and she subsequently had pembrolizumab single-agent immunotherapy.

## 3. Discussion

In the UK, melanoma is the 5th most common cancer with about 16,200 new cases (4% of all cancers) and 1.0% of cancer deaths [1]. About a third of the cases may develop metastasis to the lung, brain, liver, and rarely to the bladder where it could remain undiagnosed except when the patient develops lower urinary tract symptoms [7, 8].

Those individuals with this pathology can present a wide range of symptoms, with the main presenting complaint being visible haematuria as documented in most of the reviewed cases. Other uncommon symptoms include urinary frequency, dysuria, suprapubic pain, and urinary retention [3]. A high index of suspicion should be made in patients with haematuria and a history of melanoma. Bladder metastases are usually synchronous with disseminated metastases and do confer a poor prognosis. Although the treatment options are few, the multidisciplinary team (MDT) decides on the treatment option which could vary from observation, transurethral resection, partial or radical cystectomy, radiotherapy, or immunotherapy. This is usually based on the local or systemic symptoms, patient's functional health status, invasiveness of approach, and prognosis [3–5, 7]. The initial treatment, based on the tumour size, would include a cold cup bladder biopsy or a formal transurethral resection of the bladder tumour to confirm the diagnosis and as a form of treatment.

In the past, chemotherapeutic agents of limited clinical efficacy and significant toxicity had been used in treating metastatic melanoma, resulting in a 5-year overall survival of less than 10% and a median survival of 4–12 months [9, 10]. Ipilimumab, an antibody directed toward the cytotoxic T-lymphocyte-associated antigen (CTLA-4), was the first such treatment to show an increase in overall survival [11]. On the other hand, the recent use of monoclonal antibodies against programmed cell death protein 1 (PD-1) has shown improved efficacy and a more favorable toxicity profile. Consequently, PD-1 inhibitors such as pembrolizumab or nivolumab now constitute the first-line treatment for most patients with stage IV melanoma and have been documented to dramatically improve overall survival [12].

## 4. Conclusion

Metastatic melanoma to the bladder is a rare manifestation of diffuse metastatic disease, and the clinician should be wary of the possibility of this diagnosis when evaluating patients with haematuria. A resection of the bladder lesion, while controlling the urinary symptom, is indicated for histologic diagnosis and to guide further management and prognosis. Following a MDT review, further treatment with systemic immunotherapy is most times indicated in fit patients.

## Data Availability

The data used to support the findings of this study are included within the article.
